# The Impact of Inflammation on the In Vivo Activity of the Renal Transporters OAT1/3 in Pregnant Women Diagnosed with Acute Pyelonephritis

**DOI:** 10.3390/pharmaceutics15102427

**Published:** 2023-10-05

**Authors:** Jhohann Richard de Lima Benzi, Patrícia Pereira dos Santos Melli, Geraldo Duarte, Jashvant D. Unadkat, Vera Lucia Lanchote

**Affiliations:** 1 Department of Clinical Analyses, Toxicology and Food Science, School of Pharmaceutical Sciences of Ribeirão Preto, University of São Paulo, Ribeirão Preto 14040-903, São Paulo, Brazil; jrlbenzi@uw.edu; 2 Department of Obstetrics and Gynecology, Ribeirão Preto Medical School, University of São Paulo, Ribeirão Preto 14049-900, São Paulo, Brazil; patimelli@gmail.com (P.P.d.S.M.);; 3 Department of Pharmaceutics, University of Washington, Seattle, WA 98195, USA

**Keywords:** inflammation, pharmacokinetics, OAT1, OAT3, pregnancy, pyelonephritis, furosemide, furosemide glucuronide, renal secretory clearance

## Abstract

Inflammation can regulate hepatic drug metabolism enzymes and transporters. The impact of inflammation on renal drug transporters remains to be elucidated. We aimed to quantify the effect of inflammation (caused by acute pyelonephritis) on the in vivo activity of renal OAT1/3, using the probe drug furosemide. Pregnant women (second or third trimester) received a single oral dose of furosemide 40 mg during acute pyelonephritis (Phase 1; n = 7) and after its resolution (Phase 2; n = 7; by treatment with intravenous cefuroxime 750 mg TID for 3–7 days), separated by 10 to 14 days. The IL-6, IFN-γ, TNF-α, MCP-1, and C-reactive protein plasma concentrations were higher in Phase I vs. Phase II. The pregnant women had a lower geometric mean [CV%] furosemide CLsecretion (3.9 [43.4] vs. 6.7 [43.8] L/h) and formation clearance to the glucuronide (1.1 [85.9] vs. 2.3 [64.1] L/h) in Phase 1 vs. Phase 2. Inflammation reduced the in vivo activity of renal OAT1/3 (mediating furosemide CL_secretion_) and UGT1A9/1A1 (mediating the formation of furosemide glucuronide) by approximately 40% and 54%, respectively, presumably by elevating the plasma cytokine concentrations. The dosing regimens of narrow therapeutic window OAT drug substrates may need to be adjusted during inflammatory conditions.

## 1. Introduction

Substantial evidence has indicated that inflammation plays a crucial role in the regulation of drug-metabolizing enzymes and transporters (DMET). In vitro and animal studies have demonstrated that increased concentrations of multiple cytokines, such as interleukin (IL) 6, tumor necrosis (TNF) α, and interferon (IFN) γ, can alter the expression and/or activity of DMET [1,2,3]. However, these studies have not mimicked the physiological inflammatory conditions in humans, making it difficult to translate the findings to changes in the in vivo pharmacokinetics (PK) of drugs in humans. Studies from this research group have reported that systemic inflammation resulting from rheumatoid arthritis, systemic lupus erythematosus, visceral leishmaniasis, and chronic hepatitis C alters the activity of CYP enzymes and hepatic/intestinal drug transporters [4,5,6,7,8]. However, to date, have been no studies on the impact of inflammation on the in vivo activities of the renal transporters, especially during pregnancy.

Pregnant women are a population that has been historically excluded from clinical research, even though 200 million pregnancies occur annually worldwide [9]. Recent studies have recommended the inclusion of pregnant women in clinical studies, either for the study of new drugs or the optimization of pharmacotherapy in this population [9,10,11,12]. Pregnant women are affected by diseases, such as infections, diabetes, and gestational hypertension. For example, urinary tract infections (UTIs) are the most common bacterial infections during pregnancy [13]. UTIs are defined as the presence and replication of bacteria in the urinary tract, leading to tissue damage in the urinary system. Acute pyelonephritis stands out among severe cases of UTIs, caused mainly by the intestinal flora Gram-negative bacteria *Escherichia coli*, but also by the bacteria of the Enterobacter and Proteus and the Gram-positive bacteria of the genus Streptococcus group B [13,14]. Despite a relatively low prevalence during pregnancy (1–2%), acute pyelonephritis is one of the most common causes of prenatal hospitalization, which can lead to maternal–fetal death and is the primary cause of septic shock in pregnant women [13]. The treatment of acute pyelonephritis requires hospitalization and the use of antibiotics to resolve the condition and prevent its progression to septicemia [13]. Acute pyelonephritis results in inflammation caused by pro-inflammatory cytokines. For example, in non-pregnant women with acute pyelonephritis, the plasma concentrations of the pro-inflammatory cytokines IL-6 and IL-8, TNF-α, and protein C reactive (CRP) [15,16,17] can reach from 2 to 75 times the concentrations in healthy pregnant and non-pregnant women [15,16,17].

Furosemide is a relatively old drug that was approved by the Food and Drug Administration (FDA) in 1966 and has been used as a diuretic since then. In the presence of the anti-gout drug probenecid, a known tubular drug secretion inhibitor, furosemide’s plasma exposure increases, while its total clearance (CL) and renal clearance (CL_renal_) are decreased [18]. Subsequent studies in human primary cells such as HEK293 have confirmed and characterized furosemide as a substrate for the OAT1 and OAT3 transporters, with affinity constant values (Km) of 38.9 and 21.5 μM, respectively [19]. Recently, furosemide has been used as part of cocktails of probe drugs to evaluate the in vivo activities of the renal transporters OAT1 and OAT3 in drug–drug and drug–disease interactions [20,21]. Thus, the primary goal of this study was to study the impact of inflammation caused by acute pyelonephritis (in pregnant women) on the renal secretion clearance of furosemide. Since UGT1A9 and UGT1A1 mediate the formation of furosemide glucuronide [22,23], which is excreted unchanged in the urine, our secondary goal was to evaluate the effect of inflammation on the in vivo activities of these two enzymes.

## 2. Materials and Methods

### 2.1. Clinical Study

The research protocols were conducted according to the guidelines of the Declaration of Helsinki and were approved by the Research Ethics Committee of the School of Medicine of Ribeirão Preto from the University of São Paulo (HCFMRP-USP) and the Brazilian Registry of Clinical Trials (ReBEC, http://www.ensaiosclinicos.gov.br, accessed on 1 October 2023) under the ID number RBR-4npsyxz. All the participants received a detailed explanation about the purpose of the study, its duration, the procedures, and the possible risks involved. Informed consent was obtained from all the subjects involved in the study. The participants were free to refuse to participate or withdraw their consent at any stage of the research, without penalty or prejudice to their care and/or treatment.

Pregnant women aged over 18 years diagnosed with acute pyelonephritis with indications for treatment with antibiotics were investigated. The acute pyelonephritis diagnosis was based on clinical (costovertebral angle tenderness, fever, and general malaise) and laboratory (pyuria, positive nitrite in urine, and urine culture showing at least 10,000 colony-forming units) exams. The participants were excluded from the study if they presented at least one of the following conditions: chronic renal failure, hypertensive syndromes (chronic arterial hypertension and/or pre-eclampsia), chronic fetal distress, or other inflammatory conditions. The participants were excluded from the protocol if they used drugs that inhibit OAT 1/3.

**Figure 1:**
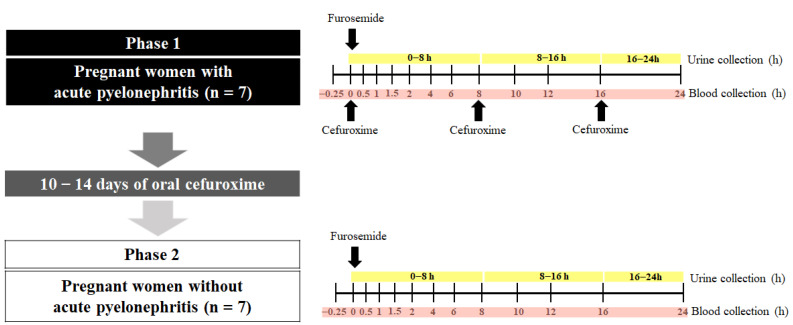
The in vivo impact of inflammation on the activity of the renal transporters OAT1/3 was quantified using a paired study design. Pregnant women diagnosed with (Phase 1) and without acute pyelonephritis (Phase 2) received a single dose of furosemide (40 mg, PO). Plasma and urine samples were collected (0–24 h). During Phase 1, acute pyelonephritis was treated with intravenous cefuroxime TID over 24 h. Phase 2 was conducted after pyelonephritis was resolved by 10–14 days of treatment with cefuroxime (250 mg/TID).

The clinical protocol was divided into 2 phases (Figure 1). In Phase 1, after the patient’s diagnosis and the indication of antibiotic treatment by the medical team, 2 mL of heparinized blood containing EDTA was collected to quantify the plasma concentration of cytokines. Anthropometric, biochemical, and hematological assessments were routinely performed by the local hospital, and such data were subsequently accessed via electronic medical records. After the administration of the first dose of antibiotic (intravenous cefuroxime, 750 mg, TID), the pregnant women received a single oral dose of 40 mg of furosemide with 200 mL of water. Serial blood samples were collected before and after the administration of furosemide at 30 min, 1; 1.5; 2; 4; 6; 8; 10; 12; 16; and 24 h [24]. The blood samples were centrifuged, and plasma was stored at −80 °C. Urine was collected over 0–24 h, the pH was immediately adjusted to 4–5 to avoid the hydrolysis of the furosemide glucuronide [25], and the volume was measured. Aliquots (10 mL) of urine were separated and stored at −80 °C. According to the local hospital protocol, after continued treatment with intravenous cefuroxime (TID for 3 to 7 days) and after showing improvement in the clinical condition, the pregnant women were discharged from the hospital and continued the treatment of oral cefuroxime (250 mg, TID) for 10–14 days.

After the end of the cefuroxime treatment, the resolution of acute pyelonephritis was confirmed, and the second phase of the protocol was carried out within the shortest possible time so that the pregnant women were in the same trimester of pregnancy as they were in Phase 1. In Phase 2, the pregnant women received a single oral dose of 40 mg of furosemide with 200 mL of water. Similar to Phase I, blood and urine samples were collected to determine the furosemide pharmacokinetics for the quantification of plasma cytokines and biochemical and hematological assessments.

### 2.2. Power Analysis

The sample size was calculated based on the furosemide pharmacokinetics in healthy volunteers administered with a single dose (40 mg, PO) of furosemide [26]. This calculation indicated that, to observe a difference of at least 40% in the renal secretion clearance (CL_secretion_) of the furosemide at *p* < 0.05 and power >80%, 7 participants would need to be studied in a pairwise fashion.

### 2.3. Analyses of Furosemide and Furosemide-Glucuronidein Plasma, Urine, and Plasma Ultrafiltrate

Furosemide and its glucuronide metabolite (FUR-GLU) concentrations in the plasma, urine, and the ultrafiltrate (from protein binding studies) were quantified using liquid chromatography coupled to tandem mass spectrometry (LC/MS), as developed and validated by us [27]. Briefly, 50 µL of plasma, urine, or ultrafiltrate were used for the analyses. The plasma samples were analyzed by acidified liquid–liquid extraction, while the urine and plasma ultrafiltrate were simply diluted with the mobile phase. The ultrafiltrate was obtained after centrifuging 200 µL of plasma through the Centrifree^®^ Ultrafiltration Device (Millipore Corp., Carrigtwohill, Ireland) as follows. The samples were centrifuged at 1875× *g* for 40 min in a centrifuge with a fixed-angle rotor (angle of 36°) (Model NT 825, Nova Técnica, Piracicaba, Brazil). The calibration lines of the total and unbound furosemide analysis were linear in the ranges of 0.50–2.500 and 0.125–250 ng/mL, respectively. Additionally, the calibration lines for the furosemide and FUR-GLU in the urine were linear in the range of 50–20,000 ng/mL. The coefficients of variation and the relative standard errors of the standard curve and quality control samples were lower than 15%.

### 2.4. Quantification of Plasma Cytokine Concentrations

The blood samples of all the participants enrolled in this study were stored at 4 °C and centrifuged (2500× *g*, 10 min, 4 °C) within 2 h of collection. The harvested plasma samples were stored at −80 °C until analysis. A broad panel of cytokines was evaluated, including IFN-γ, IL-1β, IL-2, IL-6, IL-8, IL-10, IL-12p40, IL-12p70, TNF-α, monocyte chemoattractant protein (MCP) 1, and CRP. Fifty microliters of undiluted, freshly thawed plasma and twenty-five microliters of freshly thawed plasma diluted at 1:40,000 were used for the cytokine and CRP analyses, respectively. These samples were analyzed using a 96-well plate assay, as per the manufacturer’s instructions, using the Luminex^®^ xMAP^®^ magnetic bead platform (Milliplex Map Human Cytokine Panel; Millipore, Billerica, MA, USA). Standards provided by the manufacturer were assayed in duplicates to generate calibration lines in the range from 3.2 to 10,000 pg/mL for each cytokine and from 0.01 to 50 ng/mL. The coefficients of variation and the relative standard errors of the standard curve and quality control samples were <15%.

### 2.5. Pharmacokinetic Analyses

The furosemide pharmacokinetic parameters were estimated by non-compartmental analyses using Phoenix WinNonlin^®^, version 8.3.4.295 (Certara USA, Inc., Princeton, NJ, USA, EUA). The parameters, maximum plasma concentration (C_max_), and time to C_max_ (T_max_) were documented. The area under the plasma concentration–time curve (AUC) was calculated using the linear trapezoidal rule and extrapolated to infinite by C_last_/K_el_, where C_last_ is the last predicted plasma concentration based on the terminal elimination rate (K_el_) estimated from the log-linear regression of the last four data points. The unbound fraction of furosemide (*fu*) in the plasma was determined by the ratio of the unbound plasma concentration and the total plasma concentration in the C_max_ samples (furosemide plasma protein binding has been documented to be concentration-independent) [28]. The furosemide oral clearance (CL/F) was estimated as CL/F = dose/AUC and the renal clearance (CL_renal_) was estimated as CL_renal_ = Ae/AUC_0–24h_, where Ae is the amount of furosemide excreted unchanged into the urine over 24 h (the half-life of furosemide in our study and others was 3–5 h [24,26]). The CL_secretion_ was estimated as CL_secretion_ = CL_renal_ − *fu* × creatinine clearance (CrCL), where the CrCL was estimated using the Cockcroft–Gault equation and the participant’s actual body weight [29], a recommended approach to evaluating CrCL in pregnant women. The non-renal clearance (CL/F_non-renal_) was estimated as CL/F_non-renal_ = CL/F – CL_renal_. Finally, the formation clearance to the metabolite FUR-GLU (CL_formation, FUR-GLU_) was estimated as the Ae_FUR-GLU_/AUC_0–24, furosemide_, where the Ae_FUR-GLU_ is the amount of furosemide excreted as FUR-GLU (i.e., the total amount of FUR-GLU recovered in the urine multiplied by the ratio of furosemide/FUR-GLU molecular weight). This estimation assumes that, over 24 h, most, if not all, of the metabolite formed in the body is recovered in the urine, with minimal non-renal excretion or sequential metabolism.

### 2.6. Statistical Analyses

The normality of the log-transformed data was accessed using the Shapiro–Wilk statistical test. The normally distributed parameters were compared using the Student’s *t*-test and are shown as geometric means and 90% confidence intervals, whereas the non-normally distributed parameters were compared using the Wilcoxon test and are shown as medians (interquartile ranges) [30]. In addition, the 90% confidence interval of the ratio (presence vs. absence of acute pyelonephritis) of the geometric means of the furosemide CL_renal_, CL_secretion_, CL_formation, FUR-GLU_ was computed. If this 90% confidence interval fell within the 0.8–1.25 range (i.e., the bioequivalence range), the groups were considered to be not significantly different [20]. The statistical analyses were performed using the software R (https://www.r-proje ct.org/, accessed on 1 October 2023) version 4.2.0.

## 3. Results

Seven pregnant women treated for acute pyelonephritis participated in both Phase I and II of the study. Though an additional three women participated in Phase 1, they did not participate in Phase II. Since our goal was paired comparison, they were excluded from all the data analyses. Most of the participants were in their third trimester (five out of seven; see Table 1 for the pregnant women’s demographic, biochemical, and hematological parameters). Higher median concentrations of CRP and plasma cytokines were observed during Phase I when compared to Phase II for IL-6, IFN-γ, TNF-α, and MCP-1, but not for the other cytokines (Table 2).

**Figure 2:**
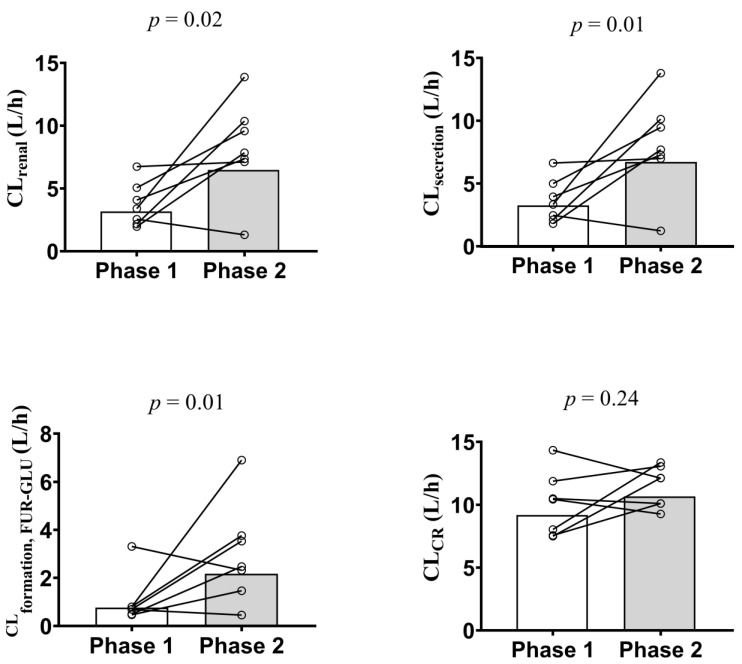
Furosemide renal (CL_renal_; **top left panel**) and secretion (CL_secretion_; **top right panel**) clearances, furosemide glucuronide formation clearance (CL_formation, FUR-GLU_; **bottom left panel**), and creatinine clearance (CLCr; **bottom right panel**) estimation in the presence (Phase 1) and absence (Phase 2) of acute pyelonephritis in 7 pregnant women after a single furosemide dose (40 mg, PO). Data are presented for each individual and the bars represent the geometric mean. The difference in the indicated parameter between Phase 1 and Phase 2 was evaluated using the paired Student’s *t*-test (*p* > 0.05). CLCr was estimated using the Cockcroft–Gault equation and the participant’s actual body weight.

**Figure 3:**
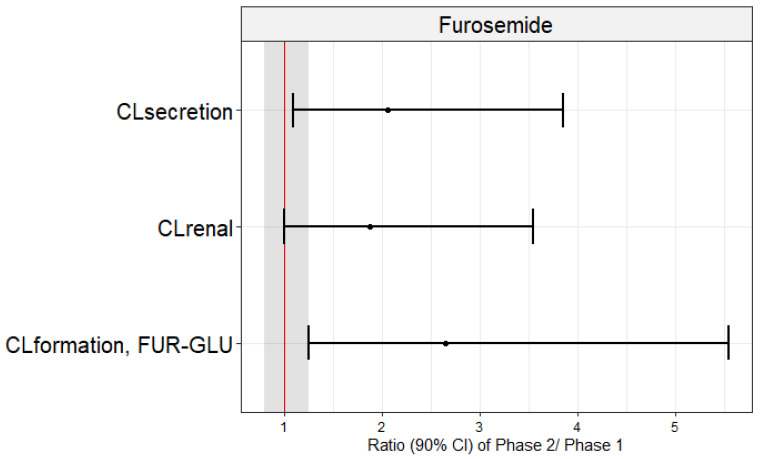
Geometric mean ratios (dots) and confidence intervals of 90% (lines) of furosemide renal (CL_renal_) and secretion (CL_secretion_) clearances and furosemide glucuronide formation clearances (CL_formation, FUR-GLU_) in pregnant women in the presence (Phase 1) and absence (Phase 2) of acute pyelonephritis. These mean ratios did not fall within the bioequivalence range (0.80–1.25; shaded area).

The geometric means of CL_renal_ (4.2 vs. 6.9 L/h), CL_secretion_ (3.9 vs. 6.7 L/h), and CL_formation, FUR-GLU_ (1.1 vs. 2.3 L/h) were significantly lower in Phase 1 when compared to Phase 2 (Table 3; Figure 2). This conclusion was confirmed when the Phase 2/Phase 1 geometric mean ratios of these parameters and their 90% confidence intervals were examined. None of the 90% confidence intervals fell within the bioequivalence threshold of 0.8–1.25 (Figure 3). In contrast, the T_max_, C_max_, AUC_0–24_, AUC_0–∞_, CL/F, *fu*, Ae, and CL/F_non-renal_ were not significantly different between Phase 1 and Phase 2 (Table 3). These results did not differ if the three pregnant women, previously excluded from the analyses, were included and the data were analyzed using an unpaired approach (data not shown).

## 4. Discussion

This study reported, for the first time, reductions in the in vivo activities of the renal transporters OAT1/3 (~40%) and UGT1A9/1A1 (~50%) due to systemic inflammation caused by acute pyelonephritis. The advantage of using acute pyelonephritis as a model for infection is that the infection can be resolved by a short course of cephalosporin (usually cefuroxime). Thus, this allowed us to study the impact of inflammation on the renal OATs in the presence and absence of acute pyelonephritis, where the same subjects acted as their own controls. We chose to study the OAT1/3 transporters because they are involved in the renal secretion of many drugs used to treat a variety of infections that result in inflammation (e.g., pyelonephritis, sepsis, and hepatitis). Additionally, the paired study minimized the important interindividual variability in the plasma cytokine concentrations [32]. This paired design allowed us to have sufficient power to determine a significant difference in the furosemide CL_secretion_ with only seven subjects. We chose to use furosemide as a probe OAT1/3 drug because most (~65–85%) of an intravenous dose of furosemide is renally eliminated by the uptake transporters OAT1/3 and efflux transporter MRP4 [33]. A smaller fraction (~35%) [34] is metabolized (likely in the liver and kidneys) into glucuronide by the UGT1A9 isoform and, to a lesser extent, by 1A1 [23]. Less than 12% of the drug is excreted unchanged in feces [35].

Our primary endpoint was CL_secretion_, rather than other systemic parameters such CL/F, AUC, or C_max_, as these can be influenced by absorption (potentially modulated by intestinal OATP2B1, BCRP, and MRP4 [34]) and metabolic processes. We and others [26,34,36] interpreted the furosemide CL_secretion_ to reflect the in vivo activity of the renal OAT1/3 transporters. This interpretation assumes that the OAT1/3-mediated active secretion is the only rate-determining step in the furosemide CL_secretion_ and CL_renal_, since the latter approximates the former. Additionally, furosemide has been documented not to be an OAT2 substrate [37]. Since furosemide exhibits CL_secretion_ and CL_renal_ that are much smaller than the renal blood flow (Q_renal_; approximately 1.2 L/min) and its blood-to-plasma partition (B/P) value is 0.6, possible changes in Q_renal_ due to acute pyelonephritis can be disregarded as a confounding factor in the interpretation of the data. Additionally, acute pyelonephritis did not affect the *fu* of the furosemide in the plasma. Thereby, we can conclude that the renal OAT 1/3 activity was reduced by inflammation, as evidenced by the lower CL_secretion_ (~43%) and CL_renal_ (~38.5%) in Phase 1 vs. Phase 2 (Table 3; Figure 2 and Figure 3). The furosemide CL_secretion_ was estimated by its filtration CL, which, in turn, was estimated by CrCL. Though creatinine CL is routinely used to estimate GFR, it is also partially secreted by OAT2 [38]. However, we observed no change in CrCL (Table 1; Figure 2), indicating that inflammation resulting from acute pyelonephritis reduces the tubular secretion of furosemide by OAT1/3 rather than glomerular filtration.

The UGT1A9/1A1 activity was also reduced by inflammation, as evidenced by a decrease in the CL_formation, FUR-GLU_ (~54%). In contrast, inflammation did not affect the other furosemide pharmacokinetic parameters (Table 3; Supplement furosemide). The lack of change in the AUC, C_max_, or T_max_ suggested that the rate and extent of furosemide absorption were not affected by the inflammation; thus, the activities of the intestinal OATP2B1, BCRP, and MRP4, did not appear to be affected by the inflammation. Moreover, the inflammation did not appear to affect the biliary clearance of the furosemide.

Inflammation is an important component of a range of clinical conditions such as bacterial, viral, fungal, and protozoal infections, chronic diseases such as type 2 diabetes mellitus, neoplasms, and autoimmune diseases such as rheumatoid arthritis and systemic lupus erythematosus [21,39,40]. Chronic or acute inflammation can result in changes in pharmacokinetics, resulting in variability in the efficacy and toxicity of drugs [21,39,41]. For example, the total clearance of meropenem was reduced by ~30–40% in critically ill patients and CRP was identified as a covariate for this reduction [42]. Meropenem is primarily eliminated renally (70%) by OAT1/3 and multidrug resistance-associated protein (MRP)4 [42,43]. Similarly, a higher plasma exposure (~70%) to the immunosuppressive mycophenolate mofetil [44,45] (primarily metabolized by hepatic UGT1A9) was observed in transplanted patients with a cytomegalovirus infection, an inflammatory viral disease [46]. However, the literature lacks in vivo studies that have characterized the activity of the renal drug transporters in inflammatory conditions. Limited data have shown a reduction in OAT1/3 mRNA (and other renal transporters) in an experimental rat inflammation model generated with lipopolysaccharide or polyinosinic:polycytidylic [47,48].

When compared to Phase II, the Phase I participants showed higher median values of some plasma cytokines such as IL-6 (97-fold), IFN-γ (6-fold), CRP (11-fold), MCP-1 (2-fold), and TNF-α (2-fold) (Table 2). Higher or similar fold-plasma concentrations (4 to 75-fold) of IL-6 have been observed in non-pregnant women with acute pyelonephritis vs. patients with asymptomatic bacteriuria, after acute pyelonephritis treatment or healthy volunteers [15,16,17]. Yet, in those studies, the CRP and TNF-α values were similar (11.2 mg/dL and 35.0 pg/mL, respectively) in the patients before and 24 h after the acute pyelonephritis treatment to those observed in Phase 1 of our study [17]. Additionally, we reported, for the first time, that the MCP-1 plasma concentrations were elevated during acute pyelonephritis, reaching similar values to those observed in critically ill COVID-19 patients. MCP-1 is also relevant in other infectious/inflammatory diseases such as tuberculosis, inflammatory bowel disease, and rheumatoid arthritis [49]. The elevated plasma concentrations of CRP and the cytokines evaluated in the present study have also been observed in other inflammatory conditions, such as rheumatoid arthritis, systemic lupus erythematosus, visceral leishmaniasis, and COVID-19 [4,6,8,50].

The cytokines IL-6, TNF-α, and IL-1β have been associated with changes in DMET expression and activity in in vitro studies. However, these have all focused on the transporters expressed in human hepatocytes. Plated human hepatocytes treated with from 100 to 10,000 pg/mL of IL-6 (a concentration range that includes the highest concentrations observed in this study) for from 8 to 48 h resulted in the reduced expression of the mRNA of several transporters, such as P-gp, MRP2, BCRP, Na+-taurocholate co-transporting polypeptide (NTCP), organic anion transporting polypeptide (OATP)2B1, OATP1B1, OATP1B3, organic cation transport (OCT) 1, and OAT 2 [2,51,52]. To date, there have been no in vitro studies that have characterized the activities or expressions of the renal transporters in the presence of cytokines. Nevertheless, we interpret the impact of pyelonephritis on the reduced renal OAT1/3 activity as being due to the elevation in the plasma cytokine concentrations reported here.

This study has some limitations. First, we assumed that the effect of acute pyelonephritis on the furosemide CL_secretion_ was caused solely by the resulting inflammation, leading to elevations in the plasma cytokine concentrations. We found no significant correlations between the cytokine plasma concentrations and the furosemide PK parameters (data not shown), probably because of the inherent variability in cytokines’ plasma concentrations and the small sample size of the study. However, we cannot discount the possibility that other physiological changes caused by the disease (or the disease–pregnancy interaction) also contributed to the observed change. Second, we assumed that the furosemide CL_secretion_ was not rate-determined by MRP4. If it was, it is possible that the inflammation reduced the activity of one or some combination of the three renal transporters (OAT1/3 and MRP4). Third, we assumed that the administration of cefuroxime during Phase 1 did not affect the furosemide CL_secretion_. Cefuroxime, a cephalosporin antibiotic, may be an OAT1/3 substrate [53,54]. Even if it is an OAT1/3 substrate, based on the following data, we do not believe that the plasma concentrations of cefuroxime observed in the study inhibited OAT1/3. The plasma C_max_ of cefuroxime observed in this study (unbound geometric mean and [CV%] of 28.81 [30.97]) mg/L) was lower than the reported cefuroxime’s IC50 (250 mg/L) to inhibit OATs [55]. Finally, no drug–drug interaction was observed when the intravenous cefuroxime (1.5 g) was administered simultaneously with the known OAT substrate, NXY-059 [56].

## 5. Conclusions

In conclusion, the data from this paired study showed that systemic inflammation, due to a bacterial infection caused by acute pyelonephritis, reduced the in vivo activity of the renal transporters OAT1/3 and renal UGT1A9/1A1 in pregnant women by approximately 40% and 50%, respectively. This magnitude of change would necessitate adjustment in the dosing regimen of drugs that have a narrow therapeutic window and are predominately cleared by OAT1/3 and/or UGT1A9/1A1.

## Figures and Tables

**Table 1:** Clinical characteristics of the pregnant women investigated in the presence (Phase 1) and absence (Phase 2) of acute pyelonephritis.

	Reference Range #	Phase 1 (n = 7)	Phase 2 (n = 7)
Age (years)	-	24.3 (16.1)	24.3 (17.4)
Gestational age (weeks)	-	26.7 (19.2)	29.6 (17.3)
Body mass index (kg/m^2^)	-	28.7 (17.0)	29.3 (17.2)
Serum creatinine (mg/dL)	0.6–1.1	0.63 (25.1)	0.5 (23.0)
Estimated creatinine clearance * (mL/min)	>90.0	161.1 (21.3)	189.2 (14.1)
AST (U/L)	3.0–32.0	22.0 (42.1)	22.7 (162.0)
ALT (U/L)	3.0–33.0	16.0 (66.1)	24.0 (187.4)
GGT (U/L)	7.0–32.0	17.5 (66.5)	27.9 (42.8)
Total plasma proteins (g/dL)	6.1–7.90	5.9 (6.72)	6.2 (4.70)
Albumin (g/dL)	3.4–4.8	3.5 (9.31)	3.6 (8.10)
α1-Acid glycoprotein	50.0–120.0	86.9 (35.1)	65.0 (23.4)
Alkaline phosphatase (U/L)	65.0–300.0	157.1 (23.6)	169.5 (22.7)
Fasting glycemia (mg/dL)	70.0–100.0	79.8 (12.1)	76.7 (11.2)
Medications in use		cefuroxime; oseltamivir; ferrous sulfate; metamizole; tramadol; folic acid; scopolamine; tinidazole (topical); terbutaline; betamethasone; levothyroxine; ondansetron; progesterone; heparin; sulfamethoxazole; and trimethoprim	ferrous sulfate; metamizole; folic acid; miconazole (topic); levothyroxine; and heparin

Data are presented as geometric mean (coefficient of variation %). * = Creatinine clearance was estimated using the Cockcroft–Gault equation and the participant’s actual body weight; ALT: Alanine aminotransferase; AST: Aspartate aminotransferase; and GGT: Gamma-glutamyl transferase. # [31].

**Table 2:** Plasma CRP and cytokines’ concentrations in the presence (Phase 1) and absence (Phase 2) of acute pyelonephritis in pregnant women.

Cytokines (pg/mL) and CRP (mg/dL)	Phase 1 (n = 7)	Phase 2 (n = 7)	*p*-Value
IFN-γ	5.80 (5.50–9.41)	0.92 (0.73–1.91)	0.0313
IL-10	32.30 (19.79–113.80)	3.12 (1.53–49.7)	0.3125
IL-12p40	3.28 (1.17–35.32)	1.34 (1.07–17.4)	>0.999
IL-12p70	1.85 (1.04–2.37)	1.04 (1.04–1.32)	0.1563
IL-1β	1.00 (0.59–4.18)	1.10 (0.60–2.40)	>0.999
IL-2	0.76 (0.62–1.21)	0.68 (0.61–1.16)	0.6875
IL-6	34.04 (1.97–126.60)	0.21 (0.11–23.7)	0.0469
IL-8	4.70 (0.22–100.83)	0.28 (0.15–31.4)	0.8438
MCP-1	807.34 (418.20–1232.50)	373.32 (277.00–403.45)	0.0313
TNF-α	41.63 (17.28–54.15)	17.01 (13.30–21.42)	0.0313
CRP	21.54 (13.46–58.84)	2.34 (1.10–3.54)	0.0313

Data presented as median (interquartile range). CRP: C-reactive protein; IFN: interferon; IL: interleukin; MCP: Monocyte chemoattractant protein; and TNF: Tumor necrosis factor. Phases were compared using the Wilcoxon signed-rank test.

**Table 3:** Furosemide (40 mg, PO) pharmacokinetic parameters in the presence (Phase 1) and absence (Phase 2) of acute pyelonephritis in pregnant women.

	Geometric Mean (CV%)		Geometric Mean Ratios (90% CI)	*p*-Value
	Phase 1 (n = 7)	Phase 2 (n = 7)	Phase 2/Phase 1	
AUC_0–24_ (ng·h/mL)	1303.0 (38.3)	1065.0 (7.1)	0.67 (0.45–1.01)	0.2386
AUC_0–∞_ (ng·h/mL)	1373.0 (38.3)	1196.0 (14.1)	0.72 (0.48–1.08)	0.4465
CL/F (L/h)	29.1 (38.8)	37.6 (7.20)	1.61 (1.10–2.35)	0.2300
CL_renal_ (L/h)	4.2 (45.5)	6.9 (43.3)	1.89 (1.01–3.54)	0.0262
CL_secretion_ (L/h)	3.9 (43.4)	6.7 (43.8)	2.06 (1.12–3.80)	0.0126
CL_formation, FUR-GLU_ (L/h)	1.1 (85.9)	2.3 (64.1)	2.65 (1.28–5.49)	0.0161
Ae (mg)	5.5 (20.8)	7.3 (46.3)	1.29 (0.73–2.28)	0.3006
CL/F_non-renal_ (L/h)	21.5 (62.4)	29.1 (14.6)	1.65 (0.92–2.96)	0.6999
C_max_ (ng/mL)	337.2 (48.5)	377.4 (39.8)	0.95 (0.51–1.77)	0.3525
T_max_ (h)	1.5 (1.0–4.0) *	1.0 (1.0–2.0) *	0.7 (0.5–1.0)	0.0938
*fu*	0.010 (32.0)	0.011 (40.3)	1.04 (0.77–1.42)	0.6499

AUC: area under the plasma concentration–time curve; CL/F: oral clearance; CL_renal_: renal clearance; CL_secretion_: secretion clearance; CL_formation, FUR-GLU_: formation clearance to furosemide glucuronide; Ae: the amount of furosemide excreted unchanged in urine over 24 h; CL/F_non-renal_: non-renal clearance; C_max_: maximum plasma concentration; T_max_: time to observe C_max_; and *fu*: fraction unbound in the plasma. T_max_ was compared between phases using the Wilcoxon test, whereas all others were compared using the paired Student’s *t*-test. * = Median (interquartile range).

## Data Availability

Data are available on reasonable request from the corresponding author.
